# On the Unique Reactivity of Pd(OAc)_2_ with Organic Azides: Expedient Synthesis of Nitriles and Imines

**DOI:** 10.1002/cctc.201300064

**Published:** 2013-04-10

**Authors:** Laura Martínez-Sarti, Silvia Díez-González

**Affiliations:** [a] Department of Chemistry, Imperial College LondonExhibition Road, South Kensington, London SW7 2AZ (UK) E-mail: s.diez-gonzalez@imperial.ac.uk; [b] Erasmus student from the Universidad de ValenciaDoctor Moliner, 50, 46100 Burjassot (Spain)

**Keywords:** azides, nitriles, oxidation, palladium, Schiff bases

Organic azides are well-established as versatile compounds that can act as precursors of different heterocycles (triazoles, triazolines, tetrazoles, etc.) or other nitrogen-containing compounds, such as amines (Staudinger reduction, Curtius rearrangement) or imines (Schmidt rearrangement, aza-Wittig reaction).^1^ Besides the ubiquitous copper-catalysed azide–alkyne cycloaddition reaction,^2^ two applications of organic azides have recently attracted the interest of the synthetic community: 1) the preparation of aziridines through the generation of nitrenes^3^ and 2) the synthesis of nitriles. We were particularly interested in the latter application, owing to the importance of the cyano group in industry,^4^ as well as its utility as an organic synthon. Traditional cyanation methods suffer from serious drawbacks, such as the use of highly toxic reagents, the generation of stoichiometric quantities of metal waste, the need for harsh conditions, and poor functional-group tolerance.

Azides allow the cyanide-free preparation of nitriles with no elongation of the skeletal carbon chain under several conditions: strong stoichiometric oxidants (such as BF_3_,5a 2,3-dichloro-5,6-dicyano-1,4-benzoquinone (DDQ),5b,c or KI/*tert*-butyl hydroperoxide),5d CuSO_4_/phenyliodonium diacetate,^6^ CuI/*tert*-butyl hydroperoxide,^7^ or a supported Ru–OH catalyst.^8^ An earlier report, which employed palladium on charcoal with an alkyne as a hydrogen acceptor, caught our attention for its neutral conditions and its scope, which was not restricted to the formation of benzonitrile derivatives [Eq. (1)].^9^


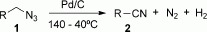
(1)

In contrast to the other methods, this Pd system required the rigorous exclusion of moisture and oxygen for any reaction to take place at reflux in benzene or diethylamine. However, despite our efforts, we could not reproduce the reported results and only conversions of <20 % were obtained. Considering that different Pd/C catalysts can lead to adventitious results, we optimised the reaction conditions (Table 1).^10^ We only obtained sluggish reactions under an inert atmosphere or in organic solvents (either anhydrous or technical-grade), but the complete conversion of compound **1 a** was achieved on water or neat (Table 1, entries 5 and 6). The reactions on water displayed poor selectivity, whereas, in the absence of solvent, anisaldehyde **4 a** (15 %) was obtained as the only by-product,^11^ although it was inseparable from compound **2 a**.

**Table 1:** Optimisation studies^[a]^

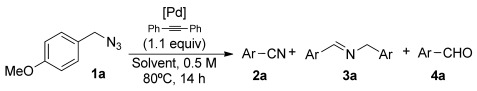						
Entry	[Pd] [mol %]	Solvent	Conversion [%]	2 a	3 a	4 a
1^[b]^	Pd/C (5)	toluene	18	55	45	0
2	Pd/C (5)	toluene	35	59	8	33
3	Pd/C (5)	dioxane	11	41	0	59
4	Pd/C (5)	MeCN	9	33	0	67
5	Pd/C (5)	water	>95	36	45	19
6	Pd/C (5)	neat	>95	85	0	15
7	[Pd_2_(dba)_3_] (5)	neat	>95	81	14	5
8	Pd(OAc)_2_ (5 or 1)	neat	>95	45	0	55
9	Pd(OAc)_2_ (1)	MeCN	>95	65	35	0
10^[c]^	Pd(OAc)_2_ (1)	MeCN	>95	84	16	0

[a] ^1^H NMR conversions are an average of two independent experiments. Pd/C was purchased from Acros Organics. [b] In anhydrous toluene under a N_2_ atmosphere. [c] The reaction was performed at a 0.12 m concentration, with styrene as a hydrogen acceptor.

If [Pd_2_(dba)_3_] was used as the catalyst (Table 1, entry 7), 1,5-diphenyl-3-pentanone was also isolated.^12^ This compound was generated by the hydrogenation of the dibenzylideneacetone (dba) ligands that were originally on the palladium centre. After screening several Pd^0^ and Pd^II^ sources, Pd(OAc)_2_ was found to be the best candidate because it suppressed the undesired formation of compound **4 a** (imine **3 a** was readily separable from compound **2 a**) and it was active in technical-grade MeCN at a concentration of only 1 mol % (Table 1, entries 6 and 10). Moreover, we also changed the hydrogen acceptor to styrene, because it is cheaper and both styrene and its reduced form can be easily removed from the crude products under reduced pressure. Notably, azide **1 a** was completely converted in the absence of styrene as well, but higher proportions of imine **3 a** and the formation of unknown by-products were observed.

Pleasantly, imines **3** were only observed as minor products in the formation of benzonitriles **2 a**–**2 c** and a diverse range of nitriles (**2**) were isolated in good-to-excellent yields. One exception was phthalimide **2 f**, which was isolated together with a bisphthalimide by-product.^12^ Also, no reaction was observed with cinnamyl azide.^13^ Terminal alkenes or carboxylic acids did not react under the optimised conditions, despite the generation of H_2_ (Table 2, entries 9 and 10). The reactions were allowed to proceed for 10 h, but shorter reaction times were sufficient in the cases of, for instance, nitriles **2 g**, **2 h**, and **2 l**.^14^

**Table 2:** Pd(OAc)_2_-catalysed synthesis of nitriles from azides

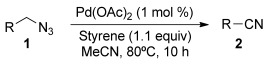			
Entry	Nitrile		Yield [%]^[a]^
1	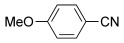	**2 a**	75
2	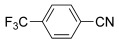	**2 b**	61
3	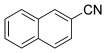	**2 c**	71
4	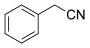	**2 d**	91
5	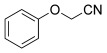	**2 e**	79
6	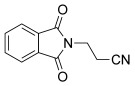	**2 f**	61^[b]^
7	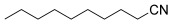	**2 g**	92
8	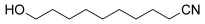	**2 h**	89
9	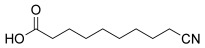	**2 i**	90
10	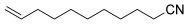	**2 j**	89
11	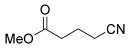	**2 k**	72
12	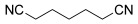	**2 l**	84^[c]^

[a] Yield of the isolated product; the values are an average of two independent experiments. Reactions were performed at a 0.12 m concentration for the formation of benzonitriles and at a 0.5 m concentration for all other substrates. [b] Isolated with *N*,*N*′-propylenebisphthalimide. [c] From 7-azidoheptanitrile.

Although organic azides are generally safe and stable towards water and oxygen,^1^ those of low molecular weight can be particularly dangerous and difficult to handle.^15^ Gratifyingly, azides **1** could be generated in situ for the one-pot formation of nitriles from their corresponding bromides and NaN_3_, thereby avoiding the need to isolate any intermediate azides (Table 3). A slightly higher palladium loading (2 mol %) and a concentration of 1 m of the starting bromide maximised the reaction conversion for a range of nitriles that contained different functional groups (Table 3).

**Table 3:** Pd(OAc)_2_-catalysed synthesis of nitriles from in situ generated azides^[a]^

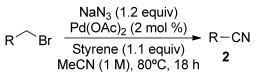			
Entry	Nitrile		Conversion [%]^[a]^
1	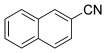	**2 c**	64^[b]^
2	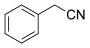	**2 d**	83
3	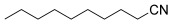	**2 g**	>95^[c]^
4	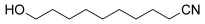	**2 h**	>95
5	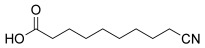	**2 i**	81
6	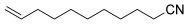	**2 j**	>95
7	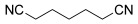	**2 l**	>95^[d]^

[a] ^1^H NMR conversions are an average of two independent experiments. [b] Imine **3 a** was also formed (36 % conversion). [c] Yield of isolated product. [d] From 7-bromoheptanitrile.

Next, two competition experiments were performed. First, we reacted cinnamyl azide **1 o** in the presence of compound **1 g** and recovered the former compound unreacted, together with 1-cyanodecane (**2 g**, Scheme 1 A). All of the previously reported catalytic systems for this transformation converted compound **1 o** into its corresponding nitrile.5b,c, 7–9 Furthermore, secondary azides have been reported to produce their corresponding ketones under the same reaction conditions as for the formation of nitriles.^11^ However, possibly owing to the absence of a strong oxidant, Pd(OAc)_2_ did not react with secondary azide **1 p**, whereas compound **1 l** was fully converted (Scheme 1 B).

**Scheme 1:**
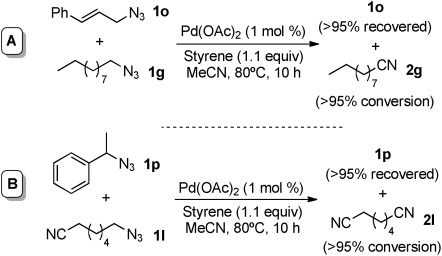
Competition experiments: A) Allyl versus alkyl azides; B) Secondary versus primary azides.

Regarding the imine formation, Pd(OAc)_2_ was also the only palladium source that was found to yield compound **3 a** as major product if the model reaction was performed under neat conditions.^10^ This result might not seem surprising, owing to the bimolecular nature of this transformation, but several Pd/C catalysts, as well as [Pd_2_(dba)_3_], actually led to the formation of nitrile **1 a** as the major product, even in the absence of solvent. Imines are often observed as minor products in the synthesis of nitriles from azides,^16^ but, to the best of our knowledge, only a molybdenum-based catalyst has been reported to produce them preferentially.^17^ As a consequence, we prepared several of these derivatives from different benzylic azides.^18^ In all cases, imines **3** were formed as the major products and they could be separated from their corresponding nitriles (**2**) by recrystallization or sublimation (Scheme 2).

**Scheme 2:**
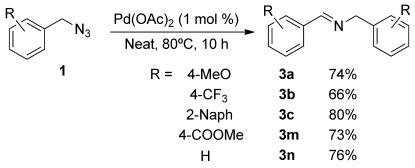
Pd(OAc)_2_-catalysed synthesis of imines from benzyl azides.

No styrene was used in the synthesis of imines because it lessened the formation of compound **3 a**, thus indicating that dihydrogen might be necessary for the imine formation. Also, nitrile **2 a** was integrally recovered after heating for 24 h at 80 °C in the presence of Pd(OAc)_2_. The reactions shown in Scheme 2 proceeded with evolution of gas within the first five minutes of stirring and the formation of ammonia was indirectly evidenced with wet pH paper. These facts lead us to propose that, in the case of aromatic nitriles, the palladium species in the reaction mixture can hydrogenate compounds **2** to generate a mixture of the imino and amino derivatives that would react together to generate compounds **3** and a molecule of ammonia (Scheme 3).^19^ Further mechanistic studies on this system are underway.

**Scheme 3:**
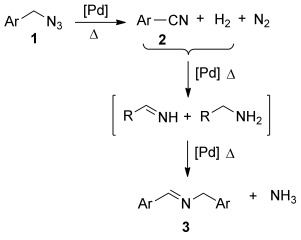
Proposed imine-formation pathway.

In conclusion, Pd(OAc)_2_ is an exceptional catalyst for the preparation of nitriles and imines from primary azides (or their corresponding bromides, thus avoiding the need to isolate the intermediary azide). The reactions proceeded under neutral conditions in air and showed unprecedented selectivities in the selected cases shown. The versatility of both azides and nitriles is expected to lead to the widespread application of this system in organic synthesis.

## Supplementary material

As a service to our authors and readers, this journal provides supporting information supplied by the authors. Such materials are peer reviewed and may be re-organized for online delivery, but are not copy-edited or typeset. Technical support issues arising from supporting information (other than missing files) should be addressed to the authors.
