# Supplementary material for: On the Unique Reactivity of Pd(OAc)2 with Organic Azides: Expedient Synthesis of Nitriles and Imines
Source: ChemCatChem. 2013 Apr 10;5(7):1722–4. doi: 10.1002/cctc.201300064 (PMC3752930; doi:10.1002/cctc.201300064)
Supplement: Supplementary file 1 [file cctc0005-1722-SD1.pdf]

Heterogeneous & Homogeneous & Bio-
CHEMCATCHEM
CATALYSIS

Supporting Information

© Copyright Wiley-VCH Verlag GmbH & Co. KGaA, 69451 Weinheim, 2013

On the Unique Reactivity of $Pd(OAc)_2$ with Organic Azides: Expedient Synthesis of Nitriles and Imines

Laura Martínez-Sarti[a, b] and Silvia Díez-González*[a]

cctc_201300064_sm_miscellaneous_information.pdf

Supporting Information

1. **General Considerations** **S2**
2. **Synthesis of Azides (1)** **S2**
3. **Optimisation Studies** **S3**
4. **Synthesis of Nitriles (2)** **S4**
5. **Synthesis of Imines (3)** **S8**
6. **NMR Spectra of New Compounds** **S10**
7. **References** **S12**

1. GENERAL CONSIDERATIONS

All reagents, including anhydrous MeCN, were used as received. Unless otherwise stated, all reactions were carried out in air and using technical solvents without any particular precautions to exclude moisture or oxygen. Columns chromatography and TLC were performed on silica gel, using UV light and a phosphomolybdic acid dip to visualize the products. ^{1}H NMR and ^{13}C NMR spectra were recorded on 400 MHz spectrometers at room temperature. Chemical shifts (δ) are reported in ppm with respect to tetramethylsilane (^{1}H NMR), or $CDCl_3$ (^{13}C NMR) as internal standards. Mass spectra (MS) were recorded on a Micromass Autospec Premier, Micromass LCT Premier or a VG Platform II spectometer using EI or ESI techniques at the Mass Spectroscopy Service of Imperial College London.

2. SYNTHESIS OF AZIDES (1)

Benzyl and alkyl azides were synthesised from the corresponding bromides by nucleophilic substitution with sodium azide in DMSO (eq. 1).[1]

$$R^1\text{—Br} \xrightarrow[\text{DMSO, RT}]{\text{NaN}_3} R^1\text{—N}_3 \qquad (1)$$

1-(Azidomethyl)-4-methoxybenzene **1a**,[2] 1-(azidomethyl)-4-(trifluoromethyl)benzene **1b**,[3] 2-(azidomethyl)naphthalene **1c**,[4] (2-azidoethyl)benzene **1d**,[5] (2-azidoethoxy)benzene **1e**,[6] *N*-(3-azidopropyl)-phthalimide **1f**,[7] 1-azidodecane **1g**,[8] 11-azido-1-undecanol **1h**,[9] 11-azidoundecanoic acid **1i**[10] 11-azidoundec-1-ene **1j**,[11] 7-azidoheptanitrile **1l**,[12] methyl 4-(azidomethyl)benzoate **1m**,[13] benzyl azide **1n**,[1] 3-(azidoprop-1-en-yl)benzene **1o**,[14] and (1-azidoethyl)benzene **1p**[1] are known in the literature and the spectroscopic data for all these compounds were in good agreement with the reported data.

Methyl 5-azidopentanoate (1k)

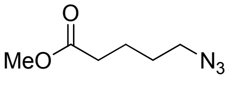

Using the general procedure reported by Alvarez from 2.15 mL g of methyl 5-bromopentanoate methyl ester, 1.34 g of the title compound were isolated (57% yield).

^{1}H NMR and mass spectra for **1k** were consistent with previously reported data for this compound.[15]

^{13}C NMR (100 MHz, $CDCl_3$): δ = 173.4 (C, *C*=O), 51.4 (CH_2, CH_2N_3), 50.9 (CH_3, OCH_3), 33.2 (CH_2), 28.1 (CH_2), 21.9 (CH_3, CH_2CH_3).

3. OPTIMISATION STUDIES

All reactions were carried out in technical solvents and in air unless stated otherwise. ^1H NMR conversions are the average of at least two independent experiments. JM stands for Johnson Matthey.

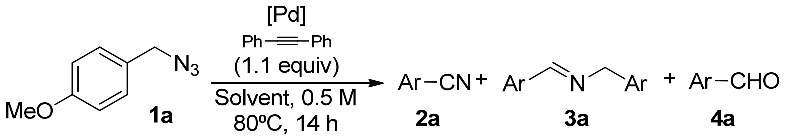

Entry	[Pd] (mol %)	Solvent	Conv (%)	2a	3a	4a
1[a]	Pd/C – Acros (5)	Toluene (0.5 M)	18	55	45	0
2	Pd/C – Acros (5)	Toluene (0.5 M)	35	59	8	33
3	Pd/C – Acros (5)	Dioxane (0.5 M)	11	41	0	59
4	Pd/C – Acros (5)	MeCN (0.5 M)	9	33	0	67
5	Pd/C – Acros (5)	*i*-PrOH (0.5 M)	26	41	7	52
6	Pd/C – Acros (5)	Water (2 mL/mmol)	>95	36	45	19
7	Pd/C – Acros (5)	Neat	>95	85	0	15
8	Pd/C (39) – JM (5)	Toluene (0.5 M)	22	43	13	54
9	Pd/C (39) – JM (5)	Dioxane (0.5 M)	10	62	0	38
10	Pd/C (39) – JM (5)	MeCN (0.5 M)	8	28	8	76
11	Pd/C (39) – JM (5)	*i*-PrOH (0.5 M)	10	27	30	73
12	Pd/C (39) – JM (5)	Water (2 mL/mmol)	>95	58	27	15
13	Pd/C (39) – JM (5)	Neat (2 mL/mmol)	91	53	25	22
14	Pd	Water (2 mL/mmol)	>95	8	82	0
15	Pd/C (395M) – JM (5)	Water (2 mL/mmol)	71	36	13	48
16	Pd/C (434) – JM (5)	Water (2 mL/mmol)	>95	50	25	25
17	Pd/Al_2O_3 (325) – JM (5)	Water (2 mL/mmol)	>95	66	31	3
18	$[PdCl_2(PPh_3)_2]$ (5)	Water (2 mL/mmol)	>95	45	42	13
19	Na_2PdCl_4 (5)	Water (2 mL/mmol)	>95	12	69	9
20	$Pd(OH)_2$(5)	Water (2 mL/mmol)	90	48	46	6
21	$PdCl_2$(5)	Water (2 mL/mmol)	>95	55	30	15
22	$Pd_2(dba)_3$(5)	Water (2 mL/mmol)	>95	70	15	15
23	Pd/C (395M) – JM (5)	Neat	>95	86	0	14
24	Pd/C (434) – JM (5)	Neat	>95	89	0	11
25	Pd/Al_2O_3 (325) – JM (5)	Neat	>95	82	16	2
26	$Pd_2(dba)_3$(5)	Neat	>95	81	14	5
27	$Pd(OAc)_2$ (5, 2 or 1)	Neat	>95	45	0	55
28	$Pd(OAc)_2$ (1)	MeCN (0.5 M)	>95	65	35	0
29	$Pd(OAc)_2$ (1)	MeCN (0.12 M)	>95	84	16	0
30	$Pd(OAc)_2$ (1)	MeCN (0.12 M)+water	>95	84	16	0

[a] Anhydrous toluene, N_2 atmosphere.

When the model reaction was carried out with $Pd_2(dba)_3$ as catalyst (23 mg, 0.05 mmol, 5 mol%, entry 26), 22 mg of 1,5-diphenyl-3-pentanone (0.09 mmol) were isolated after purification by column chromatography (pentane:diethyl ether = 90:10).
Spectroscopic data for this ketone were consistent with previously reported data for this compound.[16]

4. SYNTHESIS OF NITRILES (2)

A. General procedure for the transformation of azides into nitriles: In a 20 mL scintillation vial fitted with a screw cap, azide **1** (1.0 mmol), styrene (0.13 mL, 1.1 mmol), $Pd(OAc)_2$ (2 mg, 1 mol %) and MeCN (2 mL for alkyl azides or 8 mL for benzyl azides) were loaded. The solution was stirred at 80°C for 10 h, and then let to cool down. After filtration of the reaction mixture over a plug of celite (EtOAc), the resulting crude nitriles were purified. The reported yields are isolated yields are the average of at least two independent runs.

B. General procedure for the transformation of bromides into nitriles: In a 20 mL scintillation vial fitted with a screw cap, bromide **1** (1.0 mmol), NaN_3 (78 mg, 1.2 mmol), styrene (0.13 mL, 1.1 mmol), $Pd(OAc)_2$ (4.5 mg, 2 mol %) and MeCN (1 mL) were loaded. The solution was stirred at 80°C for 14 h, and then let to cool down. After filtration of the reaction mixture over a plug of celite (EtOAc), the reaction conversion was determined by ^1H NMR.

1-Methoxy-4-cyanobenzene (2a)

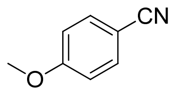

A: Using the general procedure from 153 μL of 1-(azidomethyl)-4-methoxybenzene **1a**, 0.100 g of the title compound were isolated (75% yield) after purification by sublimation at atmospheric pressure.
Spectroscopic data for **2a** were consistent with previously reported data for this compound.[17]

4-(Trifluoromethyl)benzonitrile (2b)

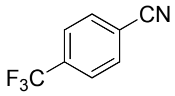

A: Using the general procedure from 153 µL of 1-(azidomethyl)-4-(trifluoromethyl)benzene **1b**, 104 mg of the title compound were isolated (61% yield) after purification by sublimation under atmospheric pressure.

Spectroscopic data for **1b** were consistent with previously reported data for this compound.[17]

2-Naphthonitrile (2c)

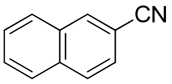

A: Using the general procedure from 183 mg of 2-(azidomethyl)naphthalene **1c**, 0.109 g of the title compound were isolated (71% yield) after recrystallisation from EtOAc (**2c** was recovered from the filtrate).

B: Using the general procedure from 0.221 g of 2-(bromomethyl)naphthalene, a total conversion of the starting bromide into nitrile **2c** and imine **3c** was observed by ^{1}H NMR (**2c**:**3c** = 64:36).

Spectroscopic data for **2c** were consistent with previously reported data for this compound.[18]

Benzyl cyanide (2d)

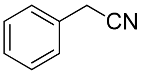

A: Using the general procedure from 0.147 g of (2-azidoethyl)benzene **1d**, 0.107 g of the title compound were isolated (79% yield) after purification by flash chromatography on silica gel (petroleum ether/diethyl ether, 90:10).

B: Using the general procedure from 0.137 mL of (2-bromoethyl)benzene, the ^{1}H NMR spectrum of the crude mixture showed 83% conversion of the starting bromide into the expected nitrile, as well as 12% of azide **1d**.

Spectroscopic data for **2d** were consistent with previously reported data for this compound.[19]

Phenoxyacetonitrile (2e)

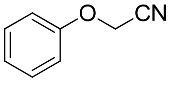

A: Using the general procedure from 0.163 g of (2-azidoethoxy)benzene **1e**, 0.106 g of the title compound were isolated (79% yield) after purification by flash chromatography on silica gel (petroleum ether/diethyl ether, 90:10).
Spectroscopic data for **2e** were consistent with previously reported data for this compound.[20]

N-(2-Cyanoethyl)-phthalimide (2f)

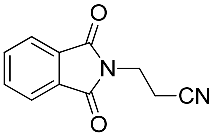

A: Using the general procedure from 0.181 g of *N*-(3-azidopropyl)-phthalimide **1f**, and after purification by flash chromatography on silica gel (petroleum ether/diethyl ether, 50:50), a fraction containing both the title compound and *N*,*N'*-propylenebisphthalimide was isolated (0.147 g, 0.61 mmol of **2f** and 0.1 mmol of the by-product according to the integration of the ^{1}H NMR spectrum) (61% yield).
Spectroscopic data for **2f** were consistent with previously reported data for this compound.[21]
N,*N'*-propylenebisphthalimide was identified by ^{1}H NMR and mass spectrometry.[22]

1-Cyanodecane (2g)

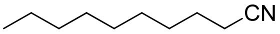

A: Using the general procedure from 0.183 g of 1-azidodecane **1g**, 0.131 g of the title compound were isolated (92% yield) after purification by flash chromatography on silica gel (petroleum ether/diethyl ether, 98:2).
B: Using the general procedure from 0.207 mL of 1-bromodecane, a total conversion into the expected nitrile was confirmed by ^{1}H NMR. The crude product was then purified by flash chromatography on silica gel (petroleum ether/diethyl ether, 98:2) to yield 0.144 g of the title compound (94% yield).
Spectroscopic data for **2g** were consistent with previously reported data for this compound.[21]

10-Cyanodecanol (2h)

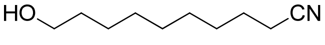

A: Using the general procedure from 0.191 g of 11-azidoundecanol **1h**, 0.245 g of the title compound were isolated (89% yield) after purification by flash chromatography on silica gel (petroleum ether/diethyl ether, 50:50).

B: Using the general procedure from 0.251 g of 11-bromoundecanol, a total conversion into the nitrile **2h** was confirmed by ^{1}H NMR.

^{1}H NMR (400 MHz, $CDCl_3$): δ = 3.59 (t, *J* = 6.0 Hz, 2H, C*H*$_2$OH), 3.31 (t, *J* = 8.0 Hz, 2H, CH_2CN), 1.90 (broad s, 1H, OH), 1.70–1.61 (m, 2H, CH_2), 1.60–1.51 (m, 2H, CH_2), 1.50–1.39 (m, 2H, CH_2), 1.39–1.20 (m, 10H); ^{13}C NMR (100 MHz, $CDCl_3$): δ = 119.8 (C, CN), 62.7 (CH_2, CH_2OH), 32.6 (CH_2, CH_2CN), 29.3 (CH_2), 29.2 (CH_2), 29.1 (CH_2), 28.6 (CH_2), 28.5 (CH_2), 25.6 (CH_2), 25.2 (CH_2), 16.7 (CH_2); HRMS (CI) calculated for $C_{11}H_{25}N_2O$ 201.1967, found 201.1960 ($[M+NH_4]^+$).

10-Cyanodecanoic acid (2i)

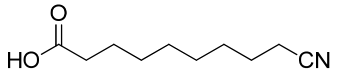

A: Using the general procedure from 0.227 g of 11-azidoundenoic acid **1i**, 0.178 g of the title compound were isolated (90% yield) after purification by flash chromatography on silica gel (EtOAc).

B: Using the general procedure from 0.265 mg of 11-bromoundenoic acid, the ^{1}H NMR spectrum of the crude mixture showed 81% conversion of the starting bromide into nitrile **2i**, as well as 19% of azide **1i**.

Spectroscopic data for **2i** were consistent with previously reported data for this compound.[23]

11-Cyanoundec-1-ene (2j)

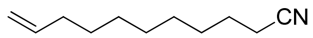

A: Using the general procedure from 0.181 g of 11-azidoundec-1-ene **1j**, 0.147 g of the title compound were isolated (89% yield) after purification by flash chromatography on silica gel (petroleum ether/diethyl ether, 95:5).

B: Using the general procedure from 0.219 mL of 11-bromoundec-1-ene, a total conversion into the expected nitrile was confirmed by ^{1}H NMR.

^{1}H NMR and mass spectra for **2j** were consistent with previously reported data for this compound.[24]

^{13}C NMR (100 MHz, $CDCl_3$): δ = 138.9 (CH, CH_2=*C*H), 119.7 (C, CN), 114.1 (CH_2, *C*H$_2$=CH), 33.6 (CH_2), 29.0 (CH_2), 28.8 (CH_2), 28.7 (CH_2), 28.6 (CH_2), 28.5 (CH_2), 25.3 (CH_2), 17.0 (CH_2).

Methyl 5-cyanopentanoate (2k)

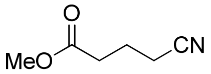

A: Using the general procedure from 0.157 g of methyl 5-azidopentanoate **1k**, 0.137 g of the title compound were isolated (72% yield) after purification by flash chromatography on silica gel (petroleum ether/diethyl ether, 50:50).

Spectroscopic data for **2k** were consistent with previously reported data for this compound.[25]

1,5-Dicyanopentane (2l)

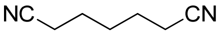

A: Using the general procedure from 0.152 g of 7-azidoheptanitrile **1l**, 0.154 g of the title compound were isolated (84% yield) after purification by flash chromatography on silica gel (petroleum ether/diethyl ether, 75:25).

B: Using the general procedure from 0.150 mL of 7-bromoheptanitrile, a 95% conversion in nitrile **2l** was confirmed by ^{1}H NMR.

^{1}H NMR (400 MHz, $CDCl_3$): δ = 2.40 (t, *J* = 6.0 Hz, 4H, C*H*$_2$CN), 1.77–1.67 (m, 4H, CH_2), 1.67–1.58 (m, 2H, CH_2); ^{13}C NMR (100 MHz, $CDCl_3$): δ = 119.2 (C, CN), 27.4 (CH_2), 24.4 (CH_2), 16.7 (CH_2); HRMS (CI) calculated for $C_7H_{14}N_3$ 140.1188, found 140.1191 ($[M+NH_4]^+$).

5. SYNTHESIS OF IMINES (3)

C. General procedure for the transformation of azides into imines: In a 20 mL thick-wall scintillation vial, azide **1** (1.0 mmol) and $Pd(OAc)_2$ (2 mg, 1 mol %) were loaded. The vial was then sealed and heated at 80°C for 10 h. The resulting crude imines were filtered over a plug of celite prior purification. The reported yields are isolated yields are the average of at least two independent runs.

***N*-(4-Methoxybenzylidene)-4-methoxybenzylamine (3a)**

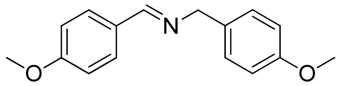

C: Using the general procedure from 153 μL of 1-(azidomethyl)-4-methoxybenzene **1a**, 92 mg of the title compound were isolated (74% yield) after recrystallisation from EtOAc.

Spectroscopic data for **3a** were consistent with previously reported data for this compound.[26]

N-(4-Trifluoromethylbenzylidene)-4-(trifluoromethyl)benzylamine (3b)

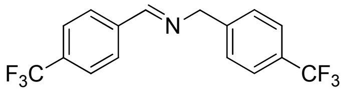

C: Using the general procedure from 0.201 g of 1-(azidomethyl)-4-(trifluoromethyl)benzene **1b**, 0.109 g of the title compound were isolated (66% yield) after purification recrystallisation from EtOAc.

Spectroscopic data for **3b** were consistent with previously reported data for this compound.[26]

N-(2'-Naphthylidene)-(2-naphthalene)methylamine (3c)

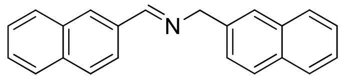

C: Using the general procedure from 0.183 g of 2-(azidomethyl)naphthalene **1c**, 0.117 g of the title compound were isolated (80% yield) after recrystallisation from EtOAc.

Spectroscopic data for **3x** were consistent with previously reported data for this compound.[27]

N-(4-Methoxycarbonylbenzylidene)-4-(methoxycarbonyl)benzylamine (3m)

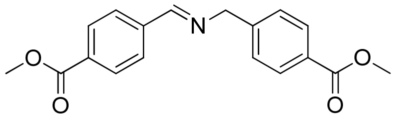

C: Using the general procedure from 0.191 g of methyl 4-(azidomethyl)benzoate **1m**, 0.113 g of the title compound were isolated (73% yield) after recrystallisation from EtOAc.

Spectroscopic data for **3m** were consistent with previously reported data for this compound.[28]

N-Benzylidenebenzylamine (3n)

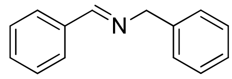

C: Using the general procedure from 125 μL of benzyl azide **1n**, 74 mg of the title compound were isolated (76% yield) after concentration under high vacuum.

Spectroscopic data for **3n** were consistent with previously reported data for this compound.[26]

6. NMR SPECTRA OF NEW COMPOUNDS

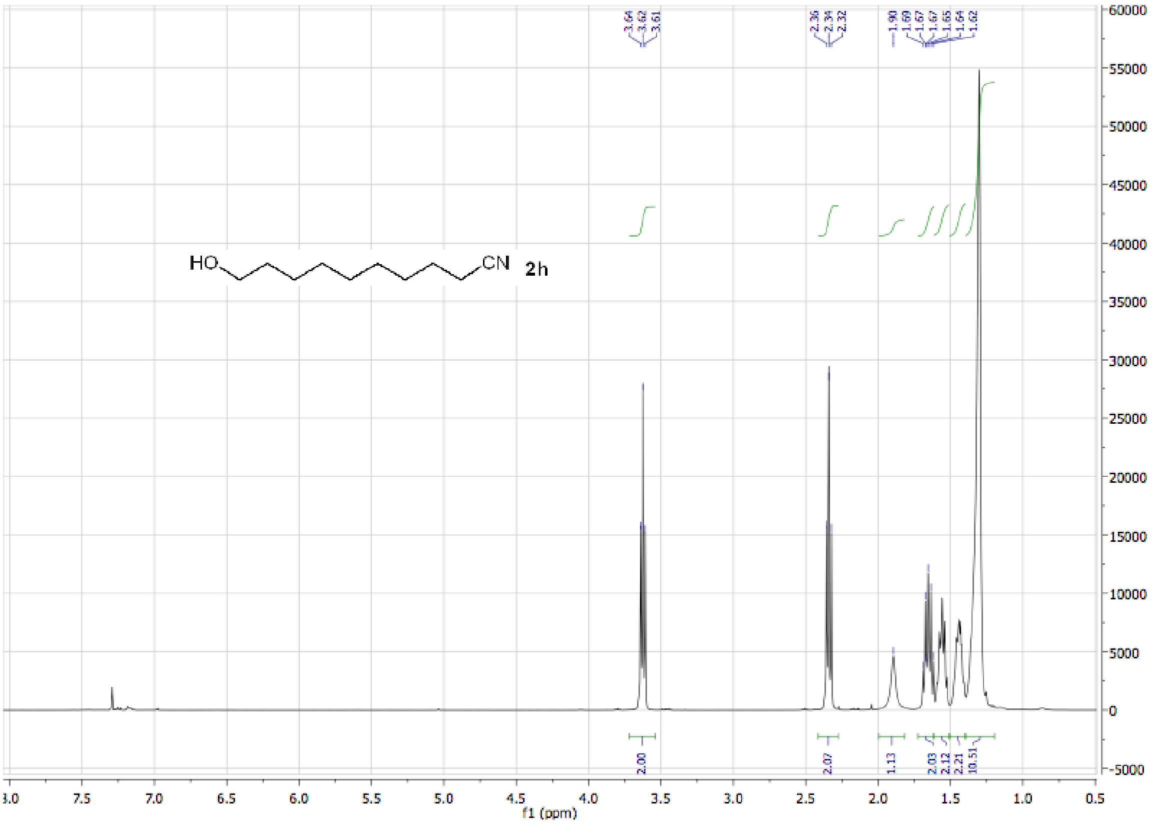

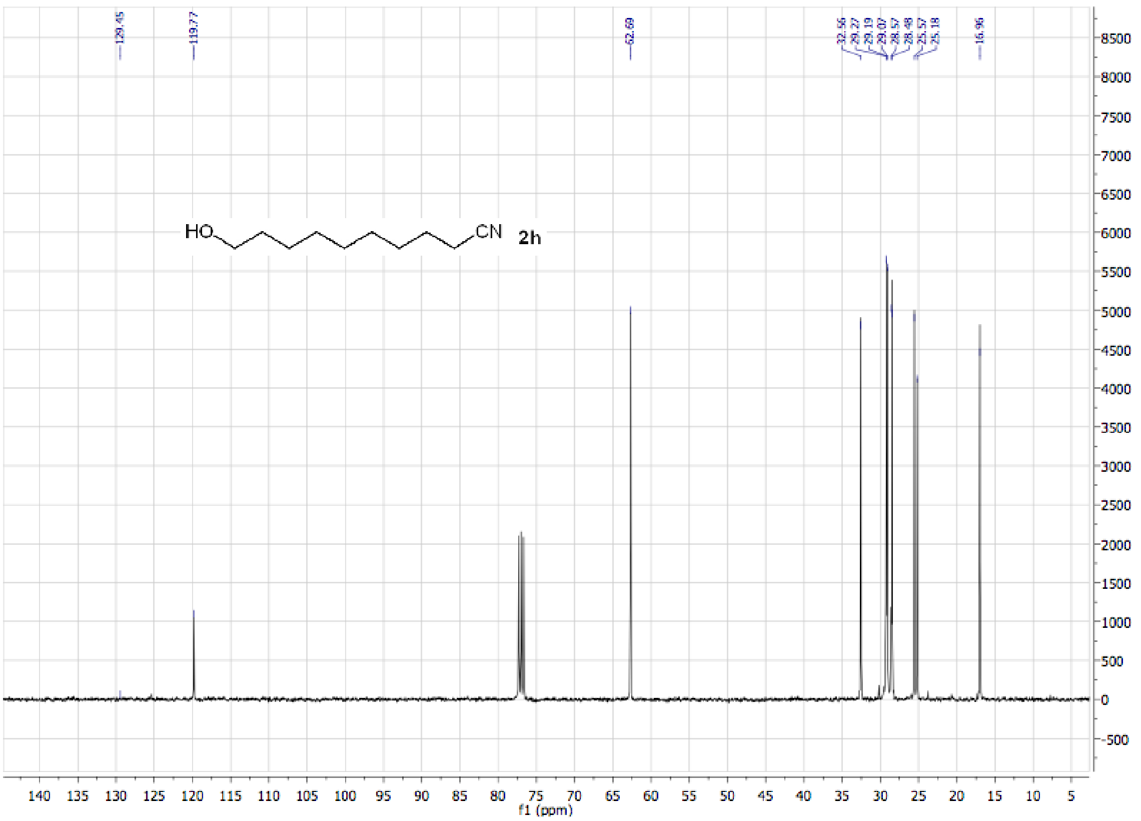

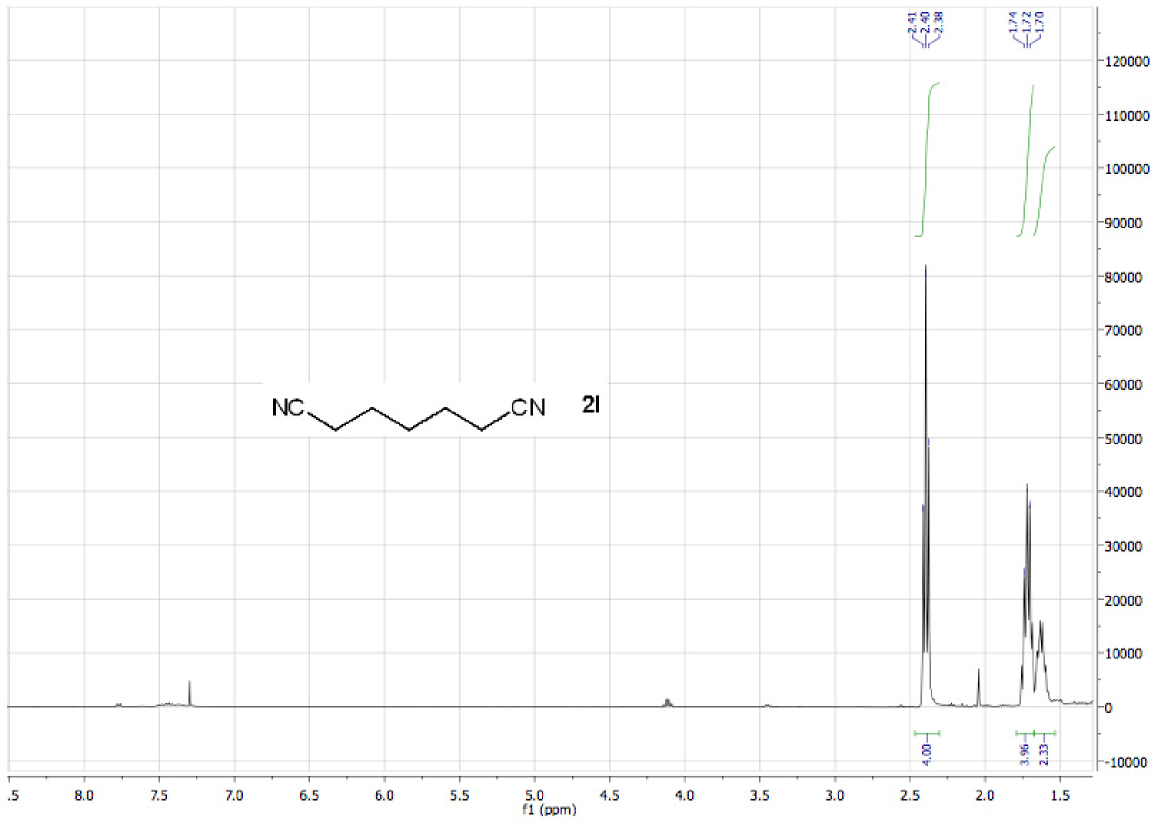

2.41
2.40
2.38
1.74
1.72
1.70
NC
CN
2l
4.00
3.96
2.33
f1 (ppm)

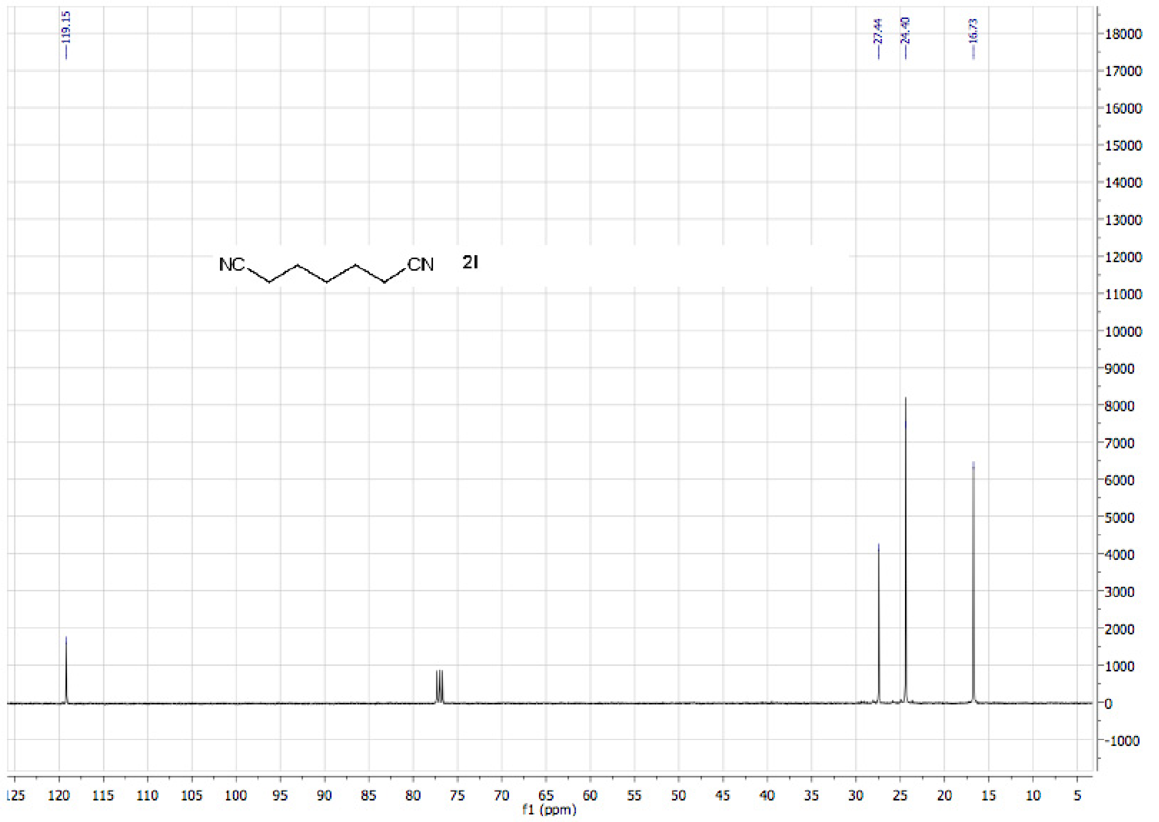

119.15
27.44
24.40
16.73
NC
CN
2l
f1 (ppm)

7. References

[1] S. G. Alvarez, M. T. Alvarez, *Synthesis* **1997**, 413–414.

[2] U. Sirion, H. J. Kim, J. H. Lee, J. W. Seo, B. S. Lee, S. J. Lee, S. J. Oh, D. Y. Chi, *Tetrahedron Lett.* **2007**, *48*, 3953–3957.

[3] H. Ankati, E. Biehl, *Tetrahedron Lett.* **2009**, *50*, 4677–4682.

[4] T. Suzuki, Y. Ota, T. Yuki, H. Tsumoto, H. Nakagawa, N. Miyata, M. Mutsuga, Y. Kawamura, M. G. Finn, *Angew. Chem.* **2010**, *122*, 6969–6972; *Angew. Chem. Int. Ed.* **2010**, ***49***, 6817–6820.

[5] M. Ito, K.-i. Koyakumaru, T. Ohta, H. Takaya, *Synthesis* **1995**, 376–378.

[6] J. Shen, R. Woodward, J. P. Kedenburg, P. G. Wang, L. Fang, D. Sun, X. Liu, M. Chen, *J. Med. Chem.* **2008**, *51*, 7417–7427.

[7] Y. Ju, D. Kumar, R. S. Varma, *J. Org. Chem.* **2006**, *71*, 6697–6700.

[8] T.-T.-T. Nguyen, F.-X. Simon, M. Schmutz, P. J. Mésini, *Chem. Commun.* **2009**, 3457–3459.

[9] S. P. Fritschi, A. Linden, H. Heimgartner, *Heterocycles* **2009**, *79*, 985–1005.

[10] C. A. Anderson, P. G. Taylor, M. A. Zeller, S. C. Zimmerman, *J. Org. Chem.* **2010**, *75*, 4848–4851.

[11] S.-C. Tsai, Y.-S. Fu, J.-H. Liao, S. J. Yu, *Helvetica Chim. Acta* **2006**, *89*, 3007–3017.

[12] L. Luo, C. Wilhelm, A. Sun, C. P. Grey, J. W. Lauher, N. S. Goroff, *J. Am. Chem. Soc.* **2008**, *130*, 7702–7709.

[13] M. Malkoch, K. Schleicher, E. Drockenmuller, C. J. Hawker, T. P. Russell, P. Wu, V. V. Fokin, *Macromolecules* **2005**, *38*, 3663–3678.

[14] H. A. van Kalkeren, J. J. Bruins, F. P. J. T. Rutjes, F. L. Van Delft, *Adv. Synth. Catal.* **2012**, *354*, 1417–1421.

[15] W. O. Moss, E. Wakefield, M. F. Mahon, K. C. Molloy, R. H. Bradbury, N. J. Hales, T. Gallagher, *Tetrahedron* **1992**, *48*, 7551–7564.

[16] G. A. Molander, L. Jean-Gérard, *J. Org. Chem.* **2009**, *74*, 1297–1303.

[17] P. Anbarasan, H. Neumann, M. Beller, *Chem. Eur. J.*, **2010**, *16*, 4725–4728.

[18] J. Kim, J. Choi, K. Shin, S. Chang, *J. Am. Chem. Soc.* **2012**, *134*, 2528–2531.

[19] R. Shang, D.-S. Ji, L. Chu, Y. Fu, L. Liu, *Angew. Chem.* **2011**, *123*, 4562–4566; *Angew. Chem. Int. Ed.* **2011**, *40*, 4470–4474.

[20] K. Wimalasena, S. W. May, *J. Am. Chem. Soc.* **1987**, *109*, 4036–4046.

[21] R. Sasson, S. Rozen, *Org. Lett.* **2005**, *7*, 2177–2179.

[22] A. Guirado, A. Zapata, M. C. Ramirez de Arellano, *Tetrahedron* **1997**, *53*, 5305–5324.

[23] S. Iida, R. Ohmura, H. Togo, *Tetrahedron* **2009**, *65*, 6257–6262.

[24] M. Imai, M. Tanaka, S. Nagumo, N. Kawahara, H. Suemune, *J. Org. Chem.* **2007**, *72*, 2543–2546.

[25] K. P Klein, T. R Demmin, B. C. Oxenrider, M. M. Rogic, M. T Tetenbaum, *J. Org. Chem.* **1979**, *44*, 275–285. See also reference 21.

[26] A. E. Wendlandt, S. Stahl, *Org. Lett.* **2012**, *14*, 2850–2853.

[27] A. Beeby, D. Parker, J. A. G. Williams, *J. Chem. Soc., Perkin Trans. 2* **1996**, 1565–1579.

[28] P. R. Ashton, P. T. Glink, J. Fraser, Stoddart, P. A. Tasker, A. J. P. White, D. J. Williams, *Chem. Eur. J.* **1996**, *2*, 729–736.
